# Force Decay Behavior of Orthodontic Elastomeric Chains in Simulated Oral Conditions

**DOI:** 10.7759/cureus.69908

**Published:** 2024-09-22

**Authors:** Gurmeet K Virdi, Anil Prashar, Gurpreet Kaur, Ravudai S Jabbal, Pancham Aggarwal, Sahil Budh, Disha Singh

**Affiliations:** 1 Orthodontics and Dentofacial Orthopedics, Desh Bhagat Dental College and Hospital, Mandi Gobindgarh, IND; 2 Prosthodontics, Genesis Institute of Dental Sciences and Research, Ferozepur, IND; 3 Orthodontics and Dentofacial Orthopedics, Advanced Dental Care Centre, Khanna, IND

**Keywords:** elastomeric chains, fluoride mouthrinse, force decay, force degradation, synthetic elastics

## Abstract

Background

Elastomeric chains are widely used for incisor retraction and space closure. However, the force they exert diminishes over time, and this makes it difficult to determine the actual force transmitted to the dentition. The force decay may also be affected by exposure to saliva, foods, beverages, and prophylactic agents.

Objective

To assess the force degradation of four commercially available orthodontic elastomeric chains (AlastiK, 3M; Generation II Power Chain, Ormco; Elastic Chain, Morelli; and Plastic Chain, American Orthodontics) over 28 days in simulated oral conditions using tooth movement and a prophylactic fluoride rinse regimen.

Method

A total of 80 chain samples, with 20 samples of each chain type having four loops each, were stretched to 25 mm on custom-fabricated acrylic resin jigs with provision for a space closure mechanism. The samples were divided equally into two groups: a control group, in which ten samples of each chain type were maintained in artificial saliva at 37°C, and a test group, in which ten samples of each chain type were maintained in artificial saliva at 37°C and additionally subjected to simulated tooth movement with jig closure of 0.25 mm/week and a twice-daily regimen of prophylactic fluoride rinse. Force decay was measured using a digital force gauge at the time of engagement on the jigs, at one hour, four hours, and at one, seven, 14, 21, and 28 days. Percentage force decay was calculated, and the results were subjected to one-way analysis of variance (ANOVA), post-hoc Tukey, post-hoc Bonferroni, and ‘t’ test.

Results

Maximum force degradation was observed at one day for all four chain materials in both groups, followed by a steady force loss over 28 days, the difference being significant for all materials at various time intervals (p<0.05). No significant difference was observed between any material at any given time in the control group (p>0.05); however, significant differences were observed in the test group. Under simulated oral conditions, force decay was maximum for the Plastic Chain, American Orthodontics (67.9 ± 6.24%), and minimum for the Generation II Power Chain, Ormco (51.9 ± 9.78%) at the end of the test period.

Conclusion

Plastic Chain, American Orthodontics, and AlastiK, 3M chain, exhibited higher force decay when compared to Generation II Power Chain, Ormco, and Elastic Chain, Morelli. Simulated oral conditions significantly affected the force decay of elastomeric chains, and it may be desirable to change elastomeric chains at 14 days for optimum retraction force.

## Introduction

Orthodontic elastomeric chains constitute an important force delivery mechanism in contemporary fixed orthodontic procedures, particularly space closure. Forces needed for space closure can also be applied using coil springs, retraction springs, loop mechanics, and magnets. Retraction springs and loops may cause discomfort due to tissue impingement and subsequent tissue trauma. Coil springs may suffer from food entrapment due to their hollow cylindrical geometry, and magnets being bulky may complicate oral hygiene procedures. Orthodontic elastomeric chains are inexpensive and easy to use, making them extremely popular auxiliaries [1].

The term ‘elastomer’ refers to polymeric materials with the ability to regain shape following the removal of an applied load [2]. This elastic behavior results from the stretching of the several cross-linkages between the main polymer chains that recoil when the applied force is removed. This behavior is exhibited till their elastic limit is achieved, beyond which permanent deformation occurs [3]. Early orthodontic elastomers were latex-based products with good elastic properties but degraded rapidly in oral environments from exposure to free radicals. Current elastomeric products are exclusively synthetic polymers based on poly(ether)urethanes or poly(ester)urethanes [4]. Several additives are incorporated by the manufacturers to improve their mechanical properties and prevent the weakening of the polymer structure by free radicals [5]. However, their exact composition remains elusive and is not disclosed by the manufacturers, accounting for variable results in laboratory testing as well as clinical outcomes.

Elastomeric chains were developed to provide light continuous forces for canine retraction, closure of diastema, generalized intra-arch space closure, and correction of tooth rotations [6]. Therefore, a sustained force application for orthodontic tooth movement is desirable, and the force degradation behavior of orthodontic elastomeric chains remains an important consideration in clinical use. Although easy to use, the elastomeric chains can absorb fluids from saliva and may leach out soluble products into the surrounding fluid medium. This can cause a breakdown of the internal structure of the polymer chain, resulting in deterioration of the mechanical properties and subsequent loss of the desirable level of force generation from the elastomeric chains [7]. This degradation of force makes it difficult to estimate the amount of force being applied by the elastomeric chain and may fail to yield the desired clinical results should the force fall below the threshold of the optimum level required for tooth movement.

Force decay in elastomeric chains is believed to occur by an initial elastic stretch followed by chain slippage. The elastic stretch involves an uncoiling of the polymer chains on load application that does not exceed the proportional limit, followed by a return to the original shape on removal of the load and is, therefore, a reversible phenomenon. However, a sustained load application causes the polymer chains to slide past one another irreversibly, producing chain slippage that is registered as a permanent deformation [8]. A multitude of factors have been shown to affect this force decay behavior including but not limited to the manufacturing process, the elastomeric chain composition, the size, and morphology of elastomeric chains, temperature, moisture, pH, tooth movement, food materials, salivary enzymes, and chemicals such as prophylactic mouth rinses [9]. 

Most investigators have reported that the rate of force loss is maximum during the first 24 hours and continues at a slow but steady rate thereafter [7,10,11]. The majority of the studies assessing the force decay characteristics of elastomeric chains have relied upon a constant stretch of the elastomeric chains, with only a few studies using simulated tooth movement [6,8,10,12,13]. Fluoride mouth rinses are frequently prescribed by orthodontists during orthodontic treatment to prevent white spot lesions and reduce the overall caries incidence. The effect of fluoride prophylactic agents has been studied, but the results reported have not been concurrent [9,14-16]. There appears to be no reported literature that assesses the force decay behavior of elastomeric chains using a combination of simulated oral conditions. The present study aimed to assess the force decay behavior of four commercially available orthodontic elastomeric chains in simulated oral conditions combining orthodontic tooth movement and prophylactic fluoride regimen at various time intervals over 28 days, the recommended time interval suggested for change of elastomeric chains.

## Materials and methods

The study was conducted at Desh Bhagat Dental College and Hospital, Punjab, India. Elastomeric chains from four manufacturers: AlastiK Chain (3M Unitek, Monrovia CA, USA), Generation II Power Chain (Ormco, Brea California, USA), Elastic Chain (Dental Morelli Ltda., Sorocaba, Sao Polo, Brazil), and Plastic Chain (American Orthodontics, Sheboygan WI, USA) with clear, closed morphology were selected for the study.

A pilot study was undertaken to determine the sample size. The power of the study was taken to be 90%, the confidence interval (CI) was taken to be 95%, and the sample size was calculated using nMaster 2.0 software (Informer Technologies, Inc., CA). Using the results from the pilot study, the sample size was estimated to be 80 for the study.

**Figure 1:**
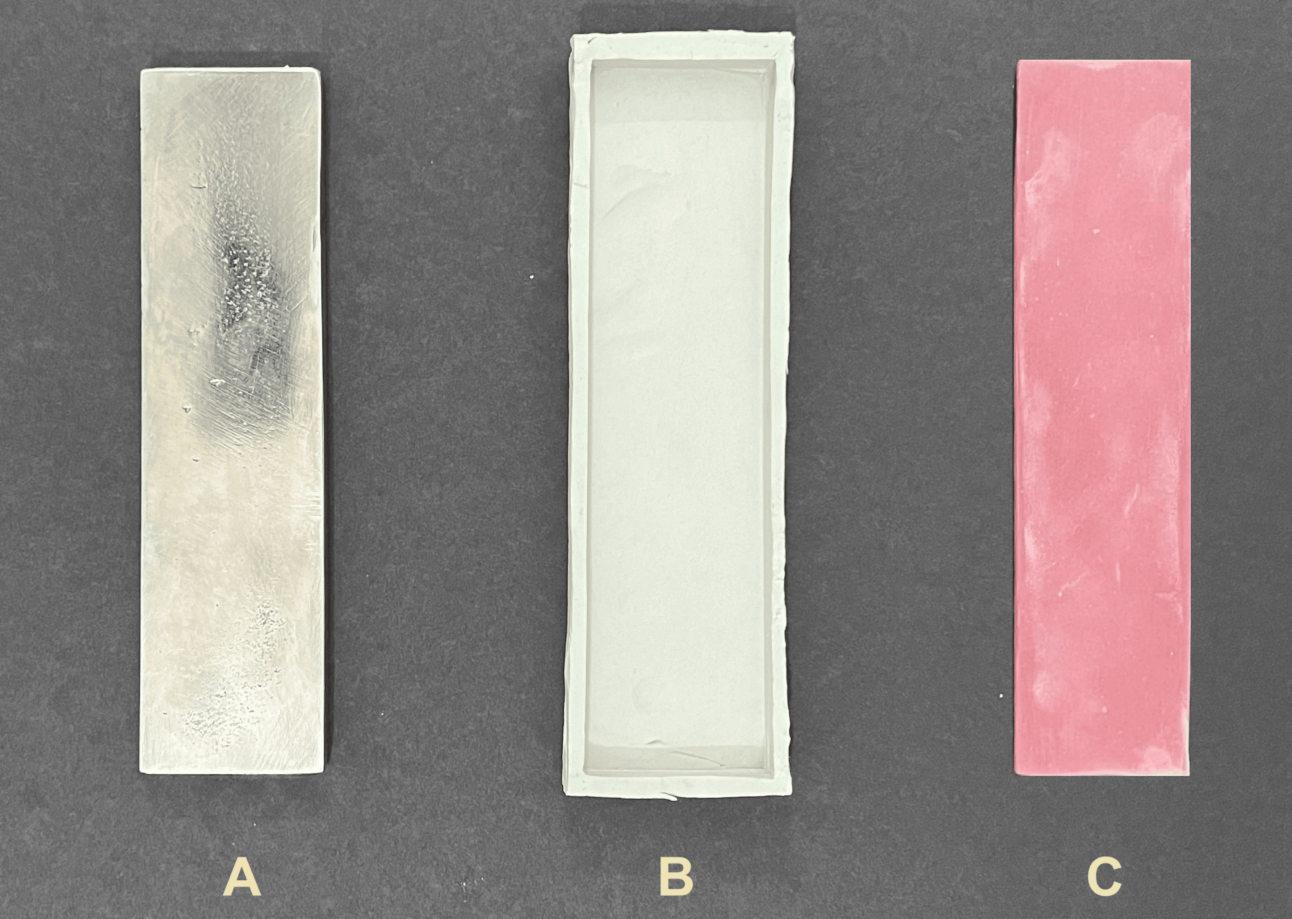
Fabrication of individual acrylic resin jig halves. A: stainless-steel mold; B: condensation silicone impression; C: acrylic resin jig half

A rectangular stainless-steel mold with dimensions 16 cm × 4 cm × 1 cm was fabricated (Figure 1A). Condensation silicone putty impression material (Speedex, Coltene/Whaledent GmbH, Langenau, Germany) was manipulated as per the manufacturer's instructions, and an impression of the stainless-steel mold was made (Figure 1B). Auto-polymerizing polymethyl methacrylate (PMMA) resin (Pyrax Polymers, Roorkee UK, India) was mixed as per the manufacturer's recommendation and poured into the set silicone impression to fabricate the acrylic resin jigs (Figure 1C). Each jig was made of two separate halves of acrylic; therefore, sixteen acrylic resin blocks were poured to fabricate eight acrylic resin jigs. The rectangular acrylic resin jigs were constructed with provisions for simulating a space closure mechanism. On both ends of each jig, a standard expansion screw (Leone SpA, Firenze, Italy) was embedded into the acrylic. The standard expansion screw served as a closing mechanism for the jigs at selected time intervals. A 2 mm space was created between the two halves of the jig by opening the expansion screw to allow for the closure of the jig halves towards each other.

**Figure 2:**
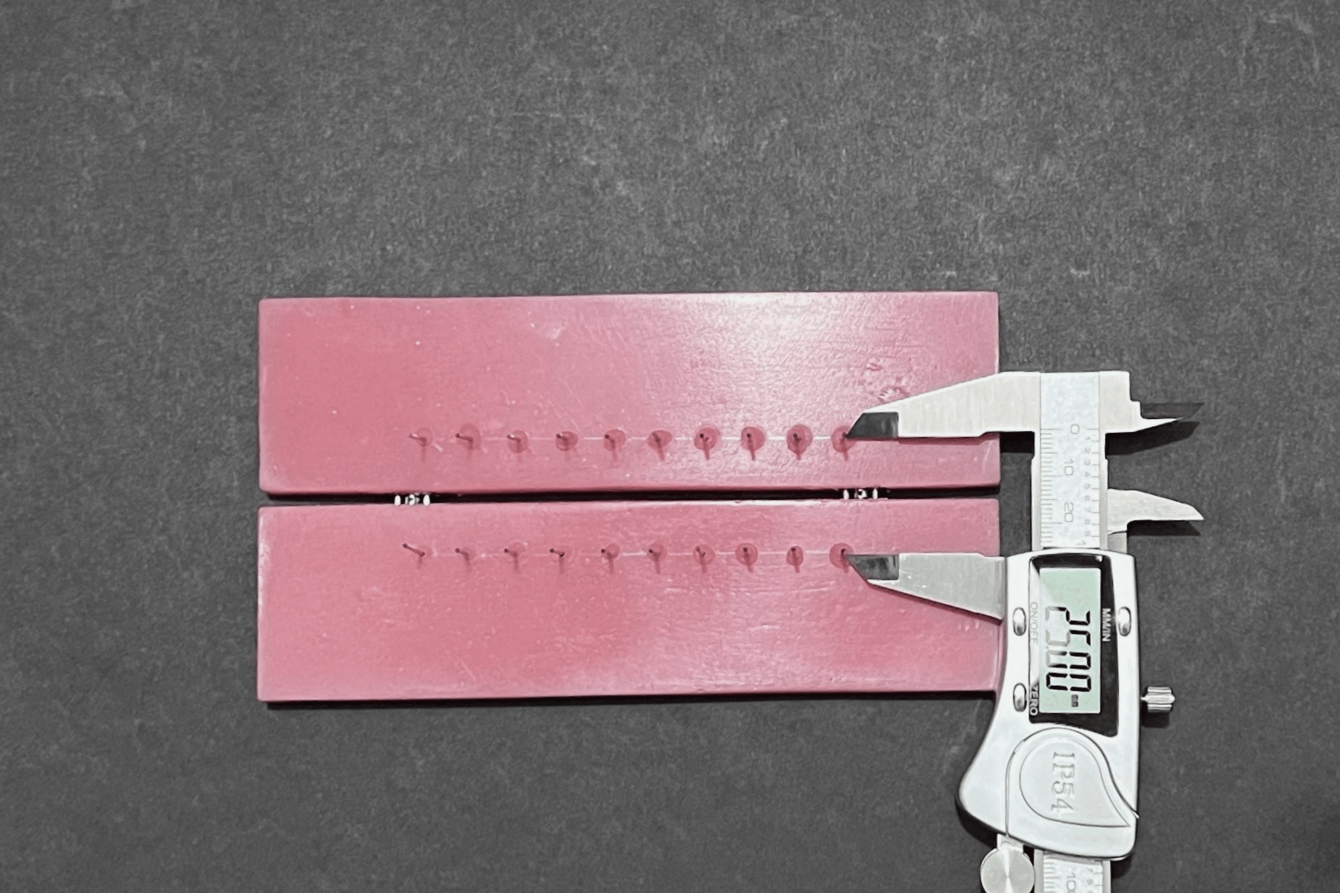
Custom acrylic jig with standard expansion screw and stainless-steel pins 25 mm apart.

Ten holes were drilled in symmetrically aligned rows on both halves of each acrylic resin jig 25 mm apart, measured using a vernier calliper (FMI, Gurugram HR, India). Stainless-steel pins, each 2 cm in length, were cut from a 0.036-inch orthodontic wire (Leone SpA, Firenze, Italy) and fixed into the holes using the auto-polymerizing PMMA resin (Figure 2). The 25 mm spacing between the stainless-steel pin pairs served as an approximation of the distance between midpoints of the first molar and canine brackets in a normal dentition before space closure.

A total of eighty samples with four loops each from the four elastomeric chains were analyzed by dividing into two groups of forty samples each. 

Control group: Consisting of forty samples from the four elastomeric chains were further subdivided as Group A_C_: 10 samples of AlastiK Chain (3M Unitek); Group O_C_: 10 samples of Generation II Power Chain (Ormco); Group M_C_: 10 samples of Elastic Chain (Dental Morelli Ltda); Group AO_C_: 10 samples of Plastic Chain (American Orthodontics).

Test group: Consisting of forty samples from the four elastomeric chains were further subdivided as Group A_T_: 10 samples of AlastiK Chain (3M Unitek); Group O_T_: 10 samples of Generation II Power Chain (Ormco); Group M_T_: 10 samples of Elastic Chain (Dental Morelli Ltda); Group AO_T_: 10 samples of Plastic Chain (American Orthodontics).

**Figure 3:**
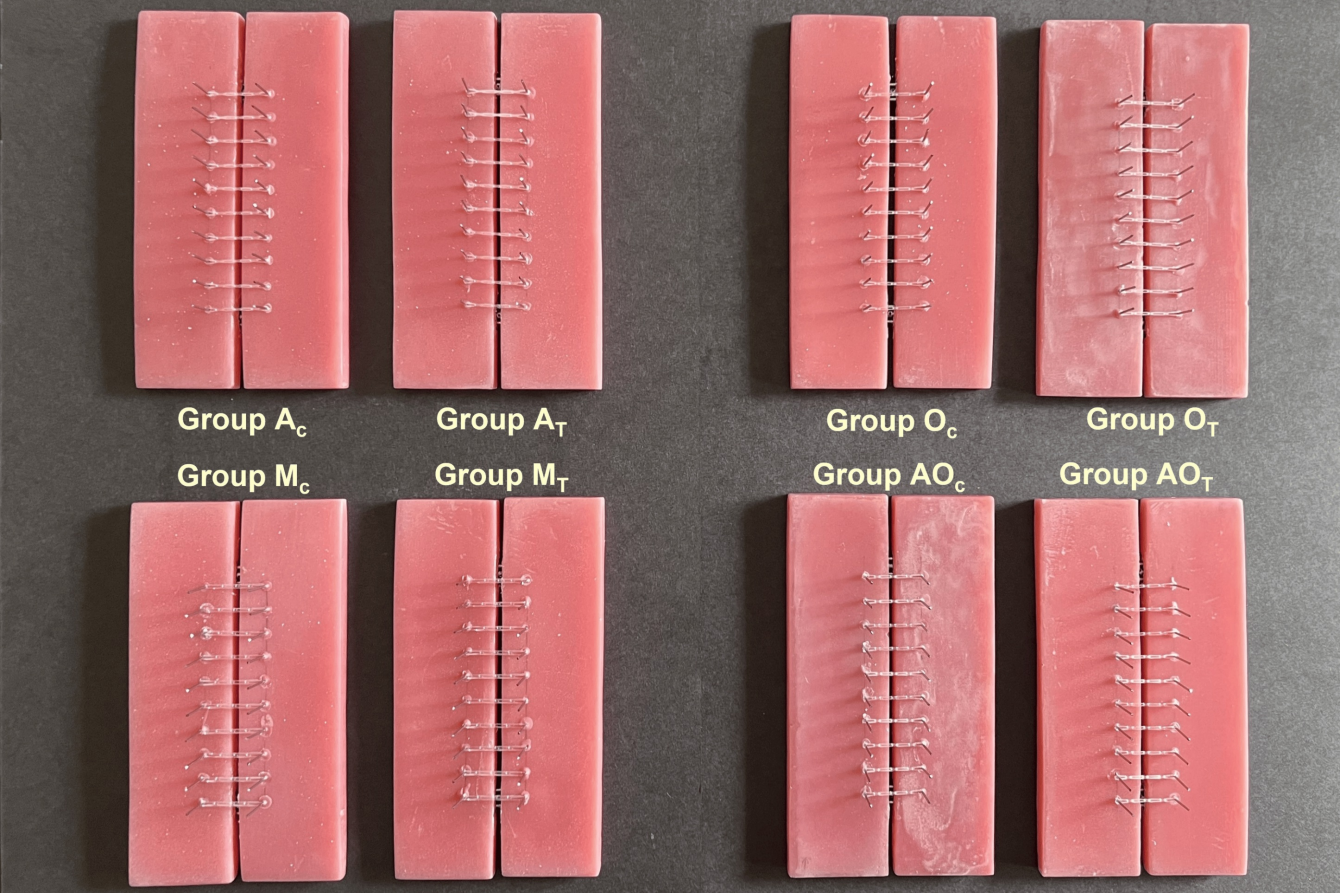
Control and test group acrylic resin jigs with elastomeric chains engaged on stainless-steel pins. Group A­­_C_: Control samples of AlastiK (3M); Group A_T_: Test samples of AlastiK (3M); Group O­­_C_: Control samples of Generation II Power Chain (Ormco); Group O_T_: Test samples of Generation II Power Chain (Ormco); Group M­­_C_: Control samples of Elastic Chain (Morelli); Group M_T_: Test samples of Elastic Chain (Morelli); Group AO­­_C_: Control samples of Plastic Chain (American Orthodontics); Group AO_T_: Test samples of Plastic Chain (American Orthodontics)

Four loops of each elastomeric chain were stretched between the two stainless-steel pins across the two halves of the acrylic resin jig, similar to what would be used clinically for space closure (Figure 3). The initial force values (grams, g) were taken for each sample for a stretch of 25 mm prior to being placed on the jigs using a digital force gauge (iScale India, Kanpur UP, India).

The samples from the control group were maintained in artificial saliva solution (Saleva, Global Dent Aids (STIM), N. Delhi, India) and stored at 37°C in an incubator to simulate oral conditions. The specimens from the test group were maintained in artificial saliva under similar conditions and were additionally exposed to 0.2% fluoride mouth rinse (ProFlo, Sandika Pharmaceuticals, N. Delhi, India) for 60 seconds twice daily. This was followed by dipping in a distilled water bath to simulate rinsing the mouth rinse from the oral cavity before being immersed again in artificial saliva.

After the initial force values were recorded before engaging the samples on the jigs, force measurements (grams, g) were recorded at one hour, four hours, one day, seven days, 14 days, 21 days, and 28 days using the digital force gauge.

For the test group, the distance between the pins was decreased by closing the standard expansion screw by 0.25 mm/week to simulate 1 mm of tooth movement over 28 days. The closures simulating tooth movement were made following force measurements for each sample of elastomeric chain at one day, seven days, 14 days, and 21 days. For the control group, the samples were kept at a 25 mm stretch throughout the study period.

**Figure 4:**
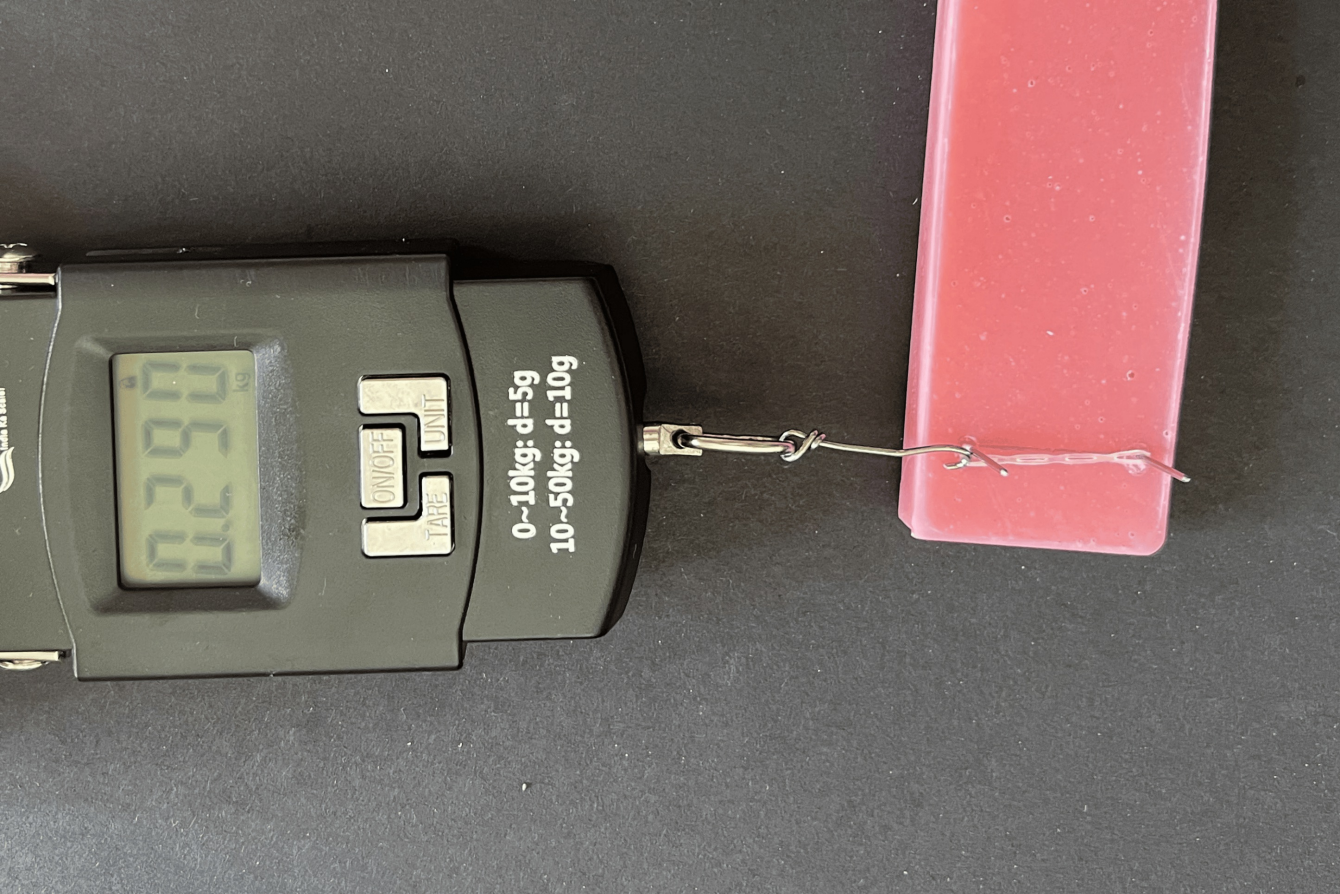
Force measurement with digital force gauge.

Force levels were determined for all samples with a 25 mm distance as standard throughout all force decay measurements. This distance was maintained by fixing two 0.036-inch stainless-steel pins on a separate acrylic resin block 25 mm apart, measured using a vernier caliper. The elastomeric chains were removed from the jigs and allowed to stabilize for five seconds before attaching to the digital force gauge. One end of each module sample was engaged on one stainless-steel pin while the other end of the module was attached to the hook of the digital force gauge and stretched until the hook was flush with the second stainless-steel pin, thus ensuring a distance of 25 mm for all force measurements (Figure 4).

The force value for all samples was recorded at the specified time intervals and force decay (expressed as a percentage) was calculated using the formula:

Force decay (%) = ((F-f)/F)×100 

Where, F: initial force before engagement of samples on resin jigs; f: force measurement at a specified time interval (one hour, four hours, one day, seven days, 14, 21, and 28 days)

The data obtained was tabulated and subjected to statistical analysis using IBM SPSS Statistics for Windows, Version 26 (Released 2019; IBM Corp., Armonk, New York, United States). All results were interpreted using a significance level of 95% (p<0.05). The data followed the normal distribution, and hence, parametric one-way analysis of variance (ANOVA) was used to determine any significant differences in the mean percentage force decay within the control and test groups at a given time interval. Subsequently, a post-hoc Tukey test was employed for intragroup pairwise comparison between any two elastomeric chain products at a specific time interval within the control and test groups. A post-hoc Bonferroni test was used for assessing intragroup pairwise inter-interval comparison for each elastomeric chain product at different time intervals within the control and test groups. Finally, the unpaired ‘t’ test was used for intergroup comparison to determine any significant differences for each elastomeric chain product between the corresponding control and test groups at similar time intervals.

## Results

For the control group, the maximum initial force was observed for Group AO_C_ (489.5 ± 39.47 g), while it was the minimum for Group A_C_ (383 ± 56.43 g). The residual force at the end of 28 days was maximum for Group M_C_ (207 ± 18.59 g) and minimum for Group A_C_ (172 ± 13.58 g). For the test group, the maximum initial force was observed for Group AO_T_ (499.5 ± 16.24 g), and the minimum initial force was observed for Group O_T_ (383.5 ± 36.52 g). The residual force at the end of 28 days was maximum for Group O_T_ (182.5 ± 13.79 g) and minimum for Group A_T_ (150.5 ± 8.64 g) (Table 1).

**Table 1:** Mean residual force (g) for the control group and test group at various time intervals. h: hour/hours; d: day/days; g: grams; SD: standard deviation

Time	0h	1h	4h	1d	7d	14d	21d	28d
CONTROL GROUP (Mean ± SD)								
Group A_C_	383±56.43	328.5±43.40	302.5±37.73	258.5±22.12	231.5±17.17	216.5±15.47	195.5±16.06	172±13.58
Group O_C_	431.5±68.19	365±21.34	337±37.87	312±39.17	275±32.66	240±35.43	211.5±35.59	188.5±30.83
Group M_C_	457±19.61	384±18.97	362.5±19.76	315±20.28	281.5±20.15	257±18.74	230±19.44	207±18.59
Group AO_C_	489.5±39.47	424±40.06	392.5±29.84	341.5±26.78	307±31.29	264±23.78	228.5±14.73	196±15.95
TEST GROUP (Mean ± SD)								
Group A_T_	443.5±71.18	289±22.46	277.5±22.64	236.5±13.34	228±10.59	203.6±8.47	172±11.11	150.5±8.64
Group O_T_	383.5±36.52	340±29.15	317±22.51	271±23.89	243±18.44	230±14.14	206±13.70	182.5±13.79
Group M_T_	430.5±40.31	366±20.79	338±25.19	285±16.50	259±15.95	217±16.53	194±13.50	169±13.90
Group AO_T_	499.5±16.24	426.5±30.65	390.5±28.33	314±20.79	279±22.34	226.5±15.10	192±14.38	160.5±14.42

The maximum mean percentage force decay for the control group was observed for Group M_C_ (15.90 ± 4.35%) at one hour, Group A_C_ (31.53 ± 9.14%) at one day, and Group AO_C_ (59.56 ± 6.15%) at 28 days. The minimum mean percentage force decay at 28 days was observed for Group A_C_ (54.38 ± 6.45%). The maximum mean percentage force decay for the test group at one hour and one day was 33.58 ± 9.81% and 45.44 ± 8.96%, respectively, for Group A_T_. At the end of 28 days, the mean percentage force decay was maximum for Group AO_T_ (67.84 ± 3.02%). Group O_T_ exhibited the least percentage of force decay at all times in the test group (Table 2). For both the test group and control group, the mean percentage force decay was greatest at one day with progressive loss up to 28 days; the mean percentage force decay was generally greater for test groups as compared to the corresponding control groups.

**Table 2:** Mean percentage (%) force decay for the control group and test group at various time intervals. h: hour/hours; d: day/days; SD: standard deviation

Time	1h	4h	1d	7d	14d	21d	28d
CONTROL GROUP (Mean ± SD)							
Group A_C_	13.65±9.33	20.21±10.28	31.53±9.14	38.37±9.97	42.31±9.63	47.95±8.79	54.38±6.45
Group O_C_	14.08±10.07	20.80±11.59	26.33±13.49	35.28±10.23	43.40±10.77	50.04±10.42	55.37±9.78
Group M_C_	15.90±4.35	20.61±4.36	31.06±3.68	38.29±5.23	43.68±4.61	49.57±4.98	54.62±4.59
Group AO_C_	13.05±8.90	19.57±5.99	29.91±6.83	36.85±8.33	45.58±7.85	52.89±6.27	59.56±6.15
TEST GROUP (Mean ± SD)							
Group A_T_	33.58±9.81	36.2±9.71	45.44±8.96	47.43±8.35	53.05±7.76	60.16±6.52	65.37±5.39
Group O_T_	10.64±10.94	16.59±10.47	28.40±11.92	35.98±8.98	39.43±7.67	45.71±7.27	51.90±6.76
Group M_T_	14.56±6.24	21.08±7.16	33.45±5.29	39.55±4.48	49.18±6.32	54.49±6.24	60.43±5.18
Group AO_T_	14.45±8.05	21.71±6.93	37.04±5.11	44.08±5.01	54.61±3.46	61.50±3.45	67.84±3.02

One-way ANOVA revealed no significant difference (p>0.05) in mean percentage force decay for the control group at any time interval; however, significant differences (p<0.05) in mean percentage force decay were observed for the test group (Table 3). A post-hoc Tukey test for intragroup pairwise material comparison of elastomeric chain products at any given time interval did not reveal any significant differences in the mean percentage force decay for the control group. For the test group, the post-hoc Tukey analysis showed that the mean percentage force decay at one hour and at four hours was significantly greater (p<0.05) for Group A_T_ as compared to Group O_T_, Group M_T_, and Group AO_T_. The mean percentage force decay at one day was significantly greater (p<0.05) for Group A_T_ as compared to Group O_T_ and Group M_T_. At seven days, the mean percentage force decay was significantly higher (p<0.05) for Group A_T_ as compared to Group O_T_. The mean percentage force decay at 14 days, 21 days, and at 28 days was significantly greater (p<0.05) for Group A_T_, Group M_T_, and Group AO_T_ as compared to Group O_T_. Also, Group M_T_ exhibited significantly lower (p<0.05) mean percentage force decay at 28 days compared to Group AO_T_ (Table 4).

**Table 3:** Overall comparison of the mean percentage force decay among different elastomeric chain products at a given time interval within the control group and test group using one-way ANOVA. h: hour/hours; d: day/days; *: significant at 95% level (p<0.05)

Time	Control Group			Test Group		
		F-value	p-value		F-value	p-value
1h	Group A_c_	0.210	0.889	Group A_T_	13.394	< 0.001*
	Group O_c_			Group O_T_		
	Group M_c_			Group M_T_		
	Group AO_c_			Group AO_T_		
4h	Group A_c_	0.040	0.989	Group A_T_	9.561	< 0.001*
	Group O_c_			Group O_T_		
	Group M_c_			Group M_T_		
	Group AO_c_			Group AO_T_		
1 d	Group A_c_	0.678	0.571	Group A_T_	7.446	0.001*
	Group O_c_			Group O_T_		
	Group M_c_			Group M_T_		
	Group AO_c_			Group AO_T_		
7 d	Group A_c_	0.283	0.838	Group A_T_	5.164	0.005*
	Group O_c_			Group O_T_		
	Group M_c_			Group M_T_		
	Group AO_c_			Group AO_T_		
14 d	Group A_c_	0.254	0.858	Group A_T_	10.860	< 0.001*
	Group O_c_			Group O_T_		
	Group M_c_			Group M_T_		
	Group AO_c_			Group AO_T_		
21 d	Group A_c_	0.679	0.571	Group A_T_	14.070	< 0.001*
	Group O_c_			Group O_T_		
	Group M_c_			Group M_T_		
	Group AO_c_			Group AO_T_		
28 d	Group A_c_	1.200	0.324	Group A_T_	17.893	< 0.001*
	Group O_c_			Group O_T_		
	Group M_c_			Group M_T_		
	Group AO_c_			Group AO_T_		

**Table 4:** Intragroup pairwise material comparison of mean percentage force decay at different time intervals using post-hoc Tukey analysis for the control group and the test group. h: hour/hours; d: day/days; *: significant at 95% level (p<0.05)

Time	Pairwise Comparison (Control)		Mean Diff	p-value	Pairwise Comparison (Test)		Mean Diff	p-value
1h	Group A_c_	Group O_c_	-0.43	1.000	Group A_T_	Group O_T_	22.95	< 0.001*
	Group A_c_	Group M_c_	-2.25	1.000	Group A_T_	Group M_T_	19.03	< 0.001*
	Group A_c_	Group AO_c_	0.60	1.000	Group A_T_	Group AO_T_	19.14	< 0.001*
	Group O_c_	Group M_c_	-1.82	1.000	Group O_T_	Group M_T_	-3.92	1.000
	Group O_c_	Group AO_c_	1.03	1.000	Group O_T_	Group AO_T_	-3.81	1.000
	Group M_c_	Group AO_c_	2.85	1.000	Group M_T_	Group AO_T_	0.11	1.000
4h	Group A_c_	Group O_c_	-0.59	1.000	Group A_T_	Group O_T_	19.61	< 0.001*
	Group A_c_	Group M_c_	-0.40	1.000	Group A_T_	Group M_T_	15.12	0.003*
	Group A_c_	Group AO_c_	0.64	1.000	Group A_T_	Group AO_T_	14.49	0.004*
	Group O_c_	Group M_c_	0.19	1.000	Group O_T_	Group M_T_	-4.49	1.000
	Group O_c_	Group AO_c_	1.22	1.000	Group O_T_	Group AO_T_	-5.12	1.000
	Group M_c_	Group AO_c_	1.04	1.000	Group M_T_	Group AO_T_	-0.63	1.000
1 d	Group A_c_	Group O_c_	5.20	1.000	Group A_T_	Group O_T_	17.04	< 0.001*
	Group A_c_	Group M_c_	0.47	1.000	Group A_T_	Group M_T_	12.00	0.016*
	Group A_c_	Group AO_c_	1.62	1.000	Group A_T_	Group AO_T_	8.40	0.181
	Group O_c_	Group M_c_	-4.72	1.000	Group O_T_	Group M_T_	-5.04	1.000
	Group O_c_	Group AO_c_	-3.58	1.000	Group O_T_	Group AO_T_	-8.64	0.155
	Group M_c_	Group AO_c_	1.15	1.000	Group M_T_	Group AO_T_	-3.60	1.000
7 d	Group A_c_	Group O_c_	3.09	1.000	Group A_T_	Group O_T_	11.45	0.005*
	Group A_c_	Group M_c_	0.07	1.000	Group A_T_	Group M_T_	7.88	0.098
	Group A_c_	Group AO_c_	1.51	1.000	Group A_T_	Group AO_T_	3.35	1.000
	Group O_c_	Group M_c_	-3.02	1.000	Group O_T_	Group M_T_	-3.57	1.000
	Group O_c_	Group AO_c_	-1.58	1.000	Group O_T_	Group AO_T_	-8.09	0.083
	Group M_c_	Group AO_c_	1.44	1.000	Group M_T_	Group AO_T_	-4.53	0.939
14 d	Group A_c_	Group O_c_	-1.07	1.000	Group A_T_	Group O_T_	13.61	< 0.001*
	Group A_c_	Group M_c_	-1.37	1.000	Group A_T_	Group M_T_	3.87	1.000
	Group A_c_	Group AO_c_	-3.27	1.000	Group A_T_	Group AO_T_	-1.56	1.000
	Group O_c_	Group M_c_	-0.29	1.000	Group O_T_	Group M_T_	-9.74	0.012*
	Group O_c_	Group AO_c_	-2.19	1.000	Group O_T_	Group AO_T_	-15.17	< 0.001*
	Group M_c_	Group AO_c_	-1.90	1.000	Group M_T_	Group AO_T_	-5.43	0.429
21 d	Group A_c_	Group O_c_	-2.09	1.000	Group A_T_	Group O_T_	14.45	< 0.001*
	Group A_c_	Group M_c_	-1.62	1.000	Group A_T_	Group M_T_	5.67	0.260
	Group A_c_	Group AO_c_	-4.95	1.000	Group A_T_	Group AO_T_	-1.34	1.000
	Group O_c_	Group M_c_	0.47	1.000	Group O_T_	Group M_T_	-8.78	0.015*
	Group O_c_	Group AO_c_	-2.86	1.000	Group O_T_	Group AO_T_	-15.79	< 0.001*
	Group M_c_	Group AO_c_	-3.32	1.000	Group M_T_	Group AO_T_	-7.01	0.083
28 d	Group A_c_	Group O_c_	-0.99	1.000	Group A_T_	Group O_T_	13.47	< 0.001*
	Group A_c_	Group M_c_	-0.24	1.000	Group A_T_	Group M_T_	4.94	0.256
	Group A_c_	Group AO_c_	-5.19	0.638	Group A_T_	Group AO_T_	-2.47	1.000
	Group O_c_	Group M_c_	0.75	1.000	Group O_T_	Group M_T_	-8.53	0.005*
	Group O_c_	Group AO_c_	-4.20	1.000	Group O_T_	Group AO_T_	-15.95	< 0.001*
	Group M_c_	Group AO_c_	-4.95	0.737	Group M_T_	Group AO_T_	-7.42	0.020*

Intragroup pairwise inter-interval comparison using the post-hoc Bonferroni test revealed significant differences in the mean percentage force decay for each elastomeric chain product compared at two different time intervals for both the control and test groups (Table 5, 6). The intergroup pairwise comparison between the control group and the test group at a given time interval for an elastomeric chain product using the unpaired ‘t’ test (Table 7) showed that mean percentage force decay values were significantly greater for Group A_T_ as compared to Group A_C_ at all time intervals, significantly greater for Group AO_T_ as compared to Group AO_C_ at all time intervals after one day, and significantly greater for Group M_T_ as compared to Group M_C_ only at 14 and 28 days, but no significant difference was observed between Group O_C_ and Group O_T_ at any time interval.

**Table 5:** Intragroup pairwise inter-interval comparison of the mean percentage force decay for each elastomeric chain product using post-hoc Bonferroni test (control group). h: hour/hours; d: day/days; *: significant at 95% level (p<0.05)

Inter-interval comparison		Group A_c_		Group O_c_		Group M_c_		Group AO_c_	
		Mean Diff	p-value	Mean Diff	p-value	Mean Diff	p-value	Mean Diff	p-value
1 h	4 h	-6.56	0.007*	-6.72	0.209	-4.71	0.198	-6.52	0.652
1 h	1 d	-17.88	< 0.001*	-12.25	0.015*	-15.15	< 0.001*	-16.86	0.002*
1 h	7 d	-24.71	0.001*	-21.20	< 0.001*	-22.39	< 0.001*	-23.80	< 0.001*
1 h	14 d	-28.66	< 0.001*	-29.31	< 0.001*	-27.78	< 0.001*	-32.53	< 0.001*
1 h	21 d	-34.30	< 0.001*	-35.96	< 0.001*	-33.67	< 0.001*	-39.84	< 0.001*
1 h	28 d	-40.73	< 0.001*	-41.29	< 0.001*	-38.72	< 0.001*	-46.51	< 0.001*
4 h	1 d	-11.32	0.002*	-5.53	0.011*	-10.44	< 0.001*	-10.34	< 0.001*
4 h	7 d	-18.15	0.004*	-14.48	0.001*	-17.68	< 0.001*	-17.28	< 0.001*
4 h	14 d	-22.10	< 0.001*	-22.59	0.002*	-23.07	< 0.001*	-26.01	< 0.001*
4 h	21 d	-27.74	< 0.001*	-29.24	< 0.001*	-28.96	< 0.001*	-33.32	< 0.001*
4 h	28 d	-34.17	< 0.001*	-34.57	< 0.001*	-34.01	< 0.001*	-39.99	< 0.001*
1 d	7 d	-6.84	0.024*	-8.95	0.023*	-7.24	0.003*	-6.94	0.012*
1 d	14 d	-10.78	< 0.001*	-17.06	0.013*	-12.63	< 0.001*	-15.67	< 0.001*
1 d	21 d	-16.42	< 0.001*	-23.71	0.001*	-18.52	< 0.001*	-22.99	< 0.001*
1 d	28 d	-22.85	< 0.001*	-29.04	< 0.001*	-23.56	< 0.001*	-29.66	< 0.001*
7 d	14 d	-3.95	0.007*	-8.11	0.108	-5.39	0.001*	-8.73	0.001*
7 d	21 d	-9.58	< 0.001*	-14.76	0.003*	-11.28	< 0.001*	-16.04	< 0.001*
7 d	28 d	-16.01	< 0.001*	-20.09	< 0.001*	-16.32	< 0.001*	-22.71	< 0.001*
14 d	21 d	-5.64	0.001*	-6.65	< 0.001*	-5.89	< 0.001*	-7.31	0.001*
14 d	28 d	-12.07	0.001*	-11.98	< 0.001*	-10.94	< 0.001*	-13.98	< 0.001*
21 d	28 d	-6.43	0.012*	-5.33	< 0.001*	-5.05	< 0.001*	-6.67	< 0.001*

**Table 6:** Intragroup pairwise inter-interval comparison of the mean percentage force decay for each elastomeric chain product using post-hoc Bonferroni test (test group). h: hour/hours; d: day/days; *: significant at 95% level (p<0.05)

Inter-interval comparison		Group A_T_		Group O_T_		Group M_T_		Group AO_T_	
		Mean Diff	p-value	Mean Diff	p-value	Mean Diff	p-value	Mean Diff	p-value
1 h	4 h	-2.62	0.040*	-5.95	0.045*	-6.52	0.033*	-7.26	0.002*
1 h	1 d	-11.86	< 0.001*	-17.77	< 0.001*	-18.89	< 0.001*	-22.60	< 0.001*
1 h	7 d	-13.85	< 0.001*	-25.35	< 0.001*	-25.00	< 0.001*	-29.63	< 0.001*
1 h	14 d	-19.47	< 0.001*	-28.80	< 0.001*	-34.62	< 0.001*	-40.16	< 0.001*
1 h	21 d	-26.58	< 0.001*	-35.07	< 0.001*	-39.94	< 0.001*	-47.05	< 0.001*
1 h	28 d	-31.79	< 0.001*	-41.26	< 0.001*	-45.87	< 0.001*	-53.40	< 0.001*
4 h	1 d	-9.25	0.001*	-11.81	0.002*	-12.37	< 0.001*	-15.34	< 0.001*
4 h	7 d	-11.23	< 0.001*	-19.39	< 0.001*	-18.48	< 0.001*	-22.37	< 0.001*
4 h	14 d	-16.85	< 0.001*	-22.85	< 0.001*	-28.10	< 0.001*	-32.90	< 0.001*
4 h	21 d	-23.96	< 0.001*	-29.12	< 0.001*	-33.42	< 0.001*	-39.79	< 0.001*
4 h	28 d	-29.17	< 0.001*	-35.31	< 0.001*	-39.35	< 0.001*	-46.13	< 0.001*
1 d	7 d	-1.99	0.001*	-7.58	0.006*	-6.11	0.016*	-7.03	0.036*
1 d	14 d	-7.60	0.007*	-11.03	0.004*	-15.73	< 0.001*	-17.56	< 0.001*
1 d	21 d	-14.71	< 0.001*	-17.31	< 0.001*	-21.05	< 0.001*	-24.45	< 0.001*
1 d	28 d	-19.93	< 0.001*	-23.49	< 0.001*	-26.98	< 0.001*	-30.80	< 0.001*
7 d	14 d	-5.62	0.022*	-3.45	0.698	-9.62	0.001*	-10.53	< 0.001*
7 d	21 d	-12.73	< 0.001*	-9.73	0.003*	-14.94	< 0.001*	-17.42	< 0.001*
7 d	28 d	-17.94	< 0.001*	-15.92	< 0.001*	-20.87	< 0.001*	-23.77	< 0.001*
14 d	21 d	-7.11	< 0.001*	-6.27	0.001	-5.32	< 0.001*	-6.89	< 0.001*
14 d	28 d	-12.32	< 0.001*	-12.46	< 0.001*	-11.25	< 0.001*	-13.24	< 0.001*
21 d	28 d	-5.21	< 0.001*	-6.19	< 0.001*	-5.93	0.001*	-6.35	< 0.001*

**Table 7:** Intergroup pairwise comparison of the mean percentage force decay for each elastomeric chain product at a given time interval using unpaired t-test. h: hour/hours; d: day/days; *: significant at 95% level (p<0.05)

Time	Group A_c _- Group A_T_		Group O_c _- Group O_T_		Group M_c _- Group M_T_		Group AO_c _- Group AO_T_	
	Mean diff	p-value	Mean diff	p-value	Mean diff	p-value	Mean diff	p-value
1 h	-19.93	0.001*	3.44	0.473	1.35	0.583	-1.39	0.717
4 h	-15.99	0.002*	4.21	0.405	-0.46	0.863	-2.13	0.514
1 d	-13.92	0.002*	-2.07	0.720	-2.39	0.256	-7.14	0.016*
7 d	-9.06	0.04*	-0.71	0.872	-1.26	0.571	-7.22	0.000*
14 d	-10.74	0.013*	3.95	0.357	-5.49	0.039*	-9.02	0.003*
21 d	-12.21	0.002*	4.33	0.295	-4.92	0.067	-8.60	0.001*
28 d	-10.99	0.000*	3.47	0.368	-5.81	0.016*	-8.28	0.001*

## Discussion

The success of orthodontic treatment depends not only on the selection of appropriate mechanics but also on the application of an optimal orthodontic force for predictable tooth movement. Elastomeric chains are commonly employed to generate forces necessary for effecting intra-arch extraction and generalized space closure, besides correction of rotations and retraction of ectopically erupted teeth into the dental arch. With improvements in material science and manufacturing techniques, contemporary elastomeric chain products have superior physical and mechanical properties that may help achieve reliable treatment outcomes. Still, exposure of elastomeric chains to an in vivo environment when in use may have a detrimental effect on their properties that can compromise the reliability as well as predictability of orthodontic treatment.

In an aqueous environment, the sorption of water can damage the polymer chains by causing the breakdown of internal bonds that can severely affect the force delivery from elastomeric chains [3]. Additionally, the application of a sustained stretch on the elastomeric chains results in load relaxation [17], a time-dependent viscoelastic phenomenon referring to the gradual loss of internal stresses within the chain structure in response to the external stretching. It has been suggested that the forces applied by the elastomeric chains follow a nonlinear progression [5], therefore the actual force being applied by the elastomeric chain is difficult to quantify accurately. Huget et al. [17] stated that the presence of ester or ether linkages increases the susceptibility of the polymer chains to hydrolysis. They suggested that exposure of synthetic elastomers to water leads to the sorption of water that acts initially as a plasticizer, causing chain slippage due to the weakening of intermolecular forces. Over time, the water sorption leads to chemical degradation of the elastomer with a reduction of load requirement. This force degradation may be hastened by cumulative effects of variations in other oral parameters such as temperature, pH, different dietary agents, and chemical ions such as fluoride present in prophylactic agents. Fluorides are routinely prescribed for their anti-cariogenic benefits during orthodontic treatment. Exposure to fluoride ions may have a role in increased force degradation of elastomeric chains [13].

The present study aimed to assess the force decay behavior of elastomeric chains with all samples maintained in artificial saliva at 37°C to simulate oral conditions. Orthodontic tooth movement was simulated with jig closure of 0.25 mm/week, and a prophylactic regimen was replicated using exposure to fluoride mouth rinse for 60 seconds twice daily. All elastomeric chains used were of the closed and clear type to ensure uniformity of color and morphology of the chain modules. Considering the ideal interval between orthodontic appointments for the replacement of elastomeric chains, the duration of this study was chosen to be four weeks.

The mean percentage force decay at one hour for the control group ranged from 13.05% (Group AO_C_) to 15.9% (Group M_C_), the difference being statistically insignificant between all groups. Balhoff et al. [8] and Mirhashemi et al. [18] reported similar values with a loss of 13%-19% and 17.93%, respectively, within the first hour. Both studies used a stretch distance of 25 mm for the elastomeric chains, similar to the present study. On the other hand, Josell et al. [1] reported higher force decay values up to 31% within the first hour using a fixed initial force of 300 g, a variable number of elastomeric chain modules, and a canine retraction distance of 28 mm. At one day, the greatest force loss was observed, with the mean percentage force decay for the control group ranging from 26.33% (Group O_C_) to 31.53% (Group M_C_). This result supports previous studies of Andreasen & Bishara [7], Weissheimer et al. [19], Masoud et al. [20], and Moghaddam et al. [21], all of whom reported that the greatest loss of force in elastomeric chains occurs within the first 24 hours but disagrees in the magnitude of force loss, with higher force decay values of 74%, 50%-55%, 30%-57%, and 30%-55%, respectively, being reported in those studies. Mirhashemi et al. [18], on the other hand, reported a similar value of force loss of 26% as observed in this study. After 28 days, the mean percentage force decay ranged from 54.38% for Group A_C_ to 59.56% for Group AO_C_. Similar results have been reported by Weissheimer et al. [19] and Omidkhoda et al. [22], with percentage force decay of 53%-61% and 51%, respectively, after 28 days in artificial saliva.

For the test group, Group A_T_ (33.58%) exhibited a significantly higher percentage of force decay as compared to Group M_T_ (14.56%), Group AO_T_ (14.45%), and Group O_T_ (10.64%) within the first hour. Balhoff et al. [8] reported a similar force decay of 13%-20% at one hour in their study using simulated tooth movement. They also reported a higher initial force loss for the AlastiK chains within the first hour, similar to what was observed for Group A_T_ in the present study, although the force decay in their study was lower at 20%. On day one, the greatest force loss was observed similar to the control group, with maximum force decay for Group A_T_ (45.44%), followed by Group AO_T_ (37.04%), Group M_T_ (33.45%), and Group O_T_ (28.40%). These results were similar to those reported by Balhoff et al. [8] and Al-Kassar [13]. At 28 days, the percentage force decay was minimum for Group O_T_ (51.90%) and maximum for Group AO_T_ (67.84%). Hershey and Reynolds [10] and Lu et al. [12] also reported similar force decay of nearly 66% and 60%-67%, respectively, after 28 days in their study. Ash and Nikolai [23] in their in vivo study also reported a comparable force decay of 72% after four weeks, implying that the force decay pattern with a 0.25 mm/week closure rate in this study closely simulated the in vivo tooth movement. Kuster et al. [24] in their combined in vitro and in vivo study stated that force decay occurs more rapidly and more extensively in intraoral use. A similar result was observed in this study, with significantly higher force decay in the test group as compared to the control group.

The present study also combined the effect of a prophylactic fluoride rinse regimen along with simulated tooth closure for a more realistic simulation of oral conditions. Omidkhoda et al. [22] reported a 23% force loss at one week and a 50% force loss at four weeks for elastomeric chains following fluoride treatment. These values were lower than those obtained in this study, probably due to the combined effect of simulated tooth movement and fluoride treatment in this study. Al-Kassar [13] using a similar closure rate of 0.5 mm/2 weeks and fluoride treatment reported a higher force loss of 69.93% after three weeks than that observed in the present study. The difference could be due to different concentrations of fluoride used in his study. Other reasons for the differing results of the present study from other comparable studies may be attributed to processing variations in module manufacturing techniques involving cutting or injection molding of the raw material, effects induced by various additives incorporated in the final product, and different morphological dimensions (ellipsoid or circular modules) or dimensional characteristics (presence or absence of inter-modular link) of the chains. The discrepancy can also result from different methodologies, such as the type of aqueous media (water, fluoride, artificial saliva) used, the amount of stretch (pre-stretched, fixed stretch, or reducing stretch), and varying intervals for force decay measurements used by different investigators.

Several clinically relevant observations were deduced from this study. Chains from Group AO_T_ (American Orthodontics) and Group A_T_ (3M) demonstrated greater percentage force decay in simulated oral conditions after 28 days, while lower percentage force decay was obtained for chains from Group M_T_ (Morelli) and Group O_T_ (Ormco). When compared to the corresponding control group, the elastomeric chains from Group A_T_ and Group AO_T_ demonstrated significantly higher force decay throughout the test period, while chains from Group M_T_ demonstrated a significant increase in percentage force decay only at the end of 28 days. The chains from Group O_T_, however, remained unaffected by the simulated oral conditions at all times and did not exhibit any significant difference from the corresponding control group.

The optimum orthodontic force required for tooth movement has been proposed by several authors to be around 250 g [25] and 300 g [26]. In the present study, the mean residual force at 28 days was maximum for Group M_C_ (207 g) and minimum for Group A_C_ (172 g) in the control group, while for the test group, the maximum and minimum mean residual force were observed for Group O_T_ (182.5 g) and Group A_T_ (150.5 g), respectively. The force remained above the optimal force till 21 days for the control group and till 14 days for the test group, implying that elastomeric chains should be replaced after two weeks of clinical use for more consistent and predictable tooth movement.

The present study being an in vitro study has some limitations because the complex in vivo environment with many variables cannot be replicated by any artificial approach. The present study was also an attempt to evaluate the cumulative effects of simulated tooth movement and a prophylactic fluoride regimen commonly prescribed during orthodontic treatment. No attempt was made to determine independently the effect of simulated tooth movement and fluoride exposure on the force decay behavior of elastomeric chains. This in vitro study can be a guide to foretell approximately the force decay characteristics of elastomeric chains in clinical usage, but the actual behavior observed in an in vivo setting may be different.

## Conclusions

Under control conditions, the maximum mean percentage force decay after 28 days was observed for Plastic Chain (American Orthodontics), followed by Generation II Power Chain (Ormco), Elastic Chain (Morelli), and AlastiK (3M). Under simulated oral conditions, the maximum mean percentage force decay after 28 days was observed for Plastic Chain (American Orthodontics), followed by AlastiK (3M), Elastic Chain (Morelli), and Generation II Power Chain (Ormco). The greatest loss of force was observed on one day for both the control group as well as the test group, followed by a steadier force loss thereafter till the end of the test period. The mean percentage force decay observed was higher for the test group as compared to the control group at all times. AlastiK (3M) and Plastic Chain (American Orthodontics) exhibited significantly higher percentage force decay under simulated mouth conditions. Generation II Power Chain (Ormco) and Elastic Chain (Morelli) behaved similarly with lower percentage force decay under simulated mouth conditions. Elastic Chain (Morelli) exhibited a significantly greater percentage of force decay only towards the end of the test period, while Generation II Power Chain (Ormco) appeared to be unaffected by the test conditions. Under simulated oral conditions, the forces generated from all the elastomeric chain products were observed to fall below the optimum threshold force required for tooth movement beyond 14 days; therefore, it may be desirable to change the elastomeric chains every two weeks for more predictable and effective tooth movement.
